# Gender stereotypes in eating disorder recognition

**DOI:** 10.1007/s40519-024-01672-6

**Published:** 2024-07-02

**Authors:** Margaret Sala, Sofia Coll, Rachel Flamer

**Affiliations:** Ferkauf Graduate School of Psychology, Yeshiva University, 1165 Morris Park Ave, Bronx, NY USA

**Keywords:** Stereotypes, Eating disorder, Anorexia nervosa, Bulimia nervosa, Binge eating disorder

## Abstract

**Purpose:**

Eating disorder (ED) awareness is low. We assessed if ED symptom recognition, perceived need for treatment, perceived distress, perceived acceptability, and perceived prevalence differed depending on the gender of the individual with the ED.

**Methods:**

276 community participants were randomly assigned to one of three gender conditions (female, male, and non-binary), read three vignettes describing three different individuals with ED symptoms [anorexia nervosa (AN), bulimia nervosa (BN), and binge eating disorder (BED)], and then answered a series of questions related to participants ED symptom recognition, perceived need for treatment, perceived distress associated with having ED symptoms, perceived acceptability (e.g., the extent to which it may not be too bad to have an ED), and perceived prevalence. Mixed ANOVAs and chi-square analyses were conducted to examine differences between groups.

**Results:**

There were no significant main effects of gender condition across the outcome variables. There were main effects of ED type for problem recognition, perceived need for treatment, perceived level of distress, and perceived prevalence, with participants being more likely to recognize a problem in the AN and BN vignettes than the BED vignettes, refer for treatment and rate a higher perceived level of distress in then AN vignette than the BN and BED vignettes, and perceive a higher prevalence rate in the BN vignette than the AN vignette. There was a significant gender by condition interaction for perceived prevalence, with participants rating a higher prevalence of AN in women and non-binary individuals than men and a higher prevalence of BN in women than non-binary individuals and men.

**Conclusion:**

These results highlight the importance of education on EDs and awareness that EDs can occur in any individual, regardless of their gender identification.

**Level of evidence:**

Level I, experimental study with randomization.

## Gender stereotypes in eating disorder recognition

One main problem with diagnosing and treating eating disorders (EDs) is that ED recognition rates are low. For example, a 2013 study by Sala and colleagues [1] found that only 41.8% of college students recognized anorexia nervosa (AN), 48.9% recognized bulimia nervosa (BN), and 24.3% recognized binge eating disorder (BED) in a series of vignettes describing individuals with EDs. Suggestion by peers that the individual seeks help from a health professional (i.e., perceived need for treatment) are also low. Sala and others [1] found that 77.1% of college students identified a probable need for treatment in the AN vignette, 37.5% in the BN vignette, and 56.5% in the BED vignette. The rates of low ED recognition and perceived need for treatment among the general population may contribute to the low treatment rates of EDs, where only 20–25% of individuals with EDs receive a diagnosis and/or seek specialized treatment [2]. Given that healthcare providers often fail to recognize EDs, it is particularly important that peers and community members are able to recognize EDs and suggest that treatment may be needed [3]. The low recognition of EDs is problematic because early detection and treatment are needed for a good ED prognosis [4], with treatment response being the greatest in the initial stages of EDs and an individual’s potential response to treatment diminishing as the disorder continues [5].

A challenge to ED recognition is the stereotypes that the general population has about who may physically look like they have an ED [6]. Some of these stereotypes may stem from how EDs are portrayed in the media as affecting White, teenage, Western women [5, 6]. Researchers have found race/ethnicity stereotypes in ED recognition, such that individuals are better able to recognize EDs in White women of higher socioeconomic status (SES) than minority women [6]. Weight stereotypes in ED recognition have also been found, with AN being more likely to be unrecognized or misdiagnosed by healthcare professionals when the presenting patient is of a higher bodyweight compared to underweight [8]. These stereotypes contribute to the low rates of ED recognition, accurate diagnoses, treatment-seeking, and perceived need for treatment in individuals who do not fit ED stereotypes [1, 5]. Although EDs occur in all groups (i.e., regardless of race/ethnicity, weight, and other types of minority status), studies have demonstrated lower rates of ED treatment among more diverse populations (e.g., individuals of non-White race ethnicity, individuals of higher weight, etc.) than among those who are White [5, 8].

However, little research to date has looked at gender stereotypes in ED recognition. While EDs are typically portrayed as affecting White women [6], EDs also occur among those identifying as men as well as non-binary individuals (i.e., individuals identifying outside of the gender binary) [10]. The DSM-5 estimates 10:1 female-to-male ratio [11] in AN. However, a 2007 study by Hudson and colleagues [12] found the number of men struggling from AN and BN to be closer to 25% of all AN and BN cases. Men with AN or BN symptomology report that they have difficulties admitting to having an ED because EDs are often seen as a woman’s problem [13]. The gender ratio for BED is even less skewed, with the prevalence for BED being 1.6% and 0.8% for women and men, respectively [11]. Prevalence rates for those identifying as non-binary are more difficult to determine due to limited research. There has been little research looking at EDs in gender nonconforming individuals, and in these studies, there has been no differentiation in prevalence rates between transgender individuals, or individuals whose gender identity is different from the sex they were assigned at birth, and those who identify as non-binary [14]. In a study examining prevalence of disordered eating in gender minorities (i.e., transgender men, transgender women, or non-binary individuals) researchers found that 26% of participants reported engaging in at least one ED behavior over the past 12 months [15]. In one study conducted at a large treatment center in the United States, 6% of adults identified as gender minorities at admission, a number that is ten times larger than that of gender minorities in the United States (0.6%) [10]. Overall, gender minority individuals appear to be at higher risk for eating disorders compared to their heterosexual peers [16] and experience perceived discrimination when seeking treatment [17].

Only a few studies to date have examined gender stereotypes in ED recognition. In one study, Schoen and colleagues found that college students were more likely to identify other specified and unspecified feeding and eating disorders in female than male characters [18]. In another study, Blackstone and colleagues found that college students were more able to identify AN and BN if the vignette target was a woman rather than a man, but that the gender of the vignette target did not impact ED recognition in BED. In another study, individuals shown the name and description of DSM-5 AN and BN rated both EDs to be feminine disorders [19]. However, these studies did not address the extent to which participants could identify EDs in non-binary individuals vs. women and men, nor differences in perceived need for treatment, perceived distress associated with having an ED, perceived acceptability (e.g., the extent to which it may not be too bad to have an ED), and perceived prevalence between women, men, and non-binary individuals. In addition to examining ED recognition, it is important to examine these other factors. Many individuals find EDs to be acceptable or desirable and not necessarily distressing, likely due to the thinness stereotypically associated with an ED [20, 21]. This is problematic, because even if an individual recognizes an ED but does not feel like it is a distressing problem, or thinks it is even desirable, it may preclude referral to treatment.

Overall, research suggests that EDs are prevalent in men, women, and individuals who identify themselves as non-binary [10, 13]. However, previous research has only examined the extent to which ED recognition differs between men vs. women. No research to date has examined the extent to which ED recognition differs in men/women vs. individuals identifying themselves as non-binary. In addition, no research to date has examined differences in perceived need for treatment, perceived distress, perceived acceptability, and perceived prevalence between women, men, and non-binary individuals. The objective of this study was to examine whether ED symptom recognition, perceived need for treatment, perceived distress, perceived acceptability, and perceived prevalence differ depending on the gender (female, male, non-binary) of the individual described. Participants read three different vignettes describing three individuals’ behaviors corresponding with symptoms of an ED (AN, BN, BED). The participants were randomly assigned to three different experimental conditions (female, male, and non-binary) of the individual in the vignette. Of note, we were unable to include a transgender condition given the difficulty of portraying it in a vignette (e.g., the inability to use pronouns to portray differences). We hypothesized that participants would be more likely to recognize the vignette description as a problem, recognize the ED symptoms, perceive a need for treatment, perceive it as more distressing, report higher acceptability, and perceive higher prevalence for: (1) Vignettes describing women than for vignettes describing individuals identifying themselves as non-binary or as men; (2) Vignettes describing individuals identifying themselves as non-binary than vignettes involving individuals identifying as men. We hypothesized that there would be no gender x diagnosis interactions.

## Methods

### Participants

Participants were recruited through listservs and social media (Twitter and Instagram). The only inclusion criteria was being 18 years of age or older. Eating disorders or gender were not mentioned in study advertisements. The initial sample consisted of 276 participants. Fifty-one participants were missing data (ranging from 13 to 89% complete). All 51 were removed from the data set because they had not responded to all three vignettes. The final sample consisted of 225 participants: 75 in the non-binary condition, 75 in the male condition, and 75 in the female condition. Participants had the opportunity to enter a drawing to win a $200 gift card for completion of the survey. The study was approved by the institutional review board and informed consent was obtained from all participants.

### Measures

*Vignette questionnaire* Participants read each of the three vignettes (AN, BN, BED) (see Appendix). These vignettes were adapted from previous studies [1] and only included symptoms that a peer may witness. Participants were then asked whether they believed the individual described in the vignette had any problems, what specific problem they had, whether they should seek treatment for the problem, how distressing the problem was, perceived acceptability of the problem, and how many others they thought struggled with the same problem. The questions and corresponding response were also adapted from previous studies [1, 5], and options were as follows:

*Problem recognition* “Do you think [NAME] has a problem?” accompanied by a 5- point Likert scale with the following answer choices: (1) Definitely Yes, (2) Probably Yes, (3) Not Sure, (4) Probably Not, and (5) Definitely Not.

*Specific problem* “What issues, if any, do you think [NAME] is struggling with?” accompanied by a free response. We chose to include this item as a free response item instead of a multiple-choice item in order to avoid influencing participants perceptions (e.g., suggesting a possible ED could make participants consider the possibility of an ED when they wouldn’t have otherwise done so). Responses were coded in the following way: (1) being able to identify any eating pathology (i.e., their responses contained key words such as anorexia, bulimia, eating disorder, binge eating disorder, self-starvation, binge/purge, eating/food problem, compulsive eating, etc.); and (2) not being able to identify any eating pathology.

*Perceived need for treatment* “Do you think [NAME] should seek help?” accompanied by a 5- point Likert scale with the following answer choices: (1) Definitely Yes, (2) Probably Yes, (3) Not Sure, (4) Probably Not, and (5) Definitely Not.

*Perceived distress* “How distressing do you think it would be to have issues similar to [NAME]?” This question assessed perceived severity and was accompanied by a 5- point Likert scale with the following answer choices: (1) Not distressing at all (2) A little distressing, (3) Distressing, (4) Very Distressing, and (5) Extremely distressing.

*Perceived acceptability* ‘‘Have you ever thought that it might not be too bad to be like [NAME]? This question assessed perceived acceptability and was accompanied by a 5- point Likert scale with the following answer choices: (1) Definitely Yes, (2) Probably Yes, (3) Not Sure, (4) Probably No, and (5) Definitely No.

*Perceived prevalence* ‘‘What percentage of individuals in the community do you think might struggle with similar issues as [NAME] at any given point in time?” This question assessed perceived prevalence and was accompanied by a visual analogue scale with a movable slider rating from (0) to (100).

### Procedure

Participants were randomly assigned to a gender condition and then completed the demographic questionnaire and the EDE-Q7. Next, participants read the vignettes corresponding to their condition (i.e., female, male, non-binary). In each condition, participants read three vignettes that described an individual with symptoms of: (1) AN; (2) BN, and (3) BED. The order of the AN, BN, and BED vignettes was randomized. Participants completed the set of vignette questions after reading each vignette. They were informed of the gender status of the participants via descriptive text and through the names of the participants and pronouns used.

### Statistical analysis

The data were screened for outliers. Outliers were defined as values that were more than 3.29 standard deviations away from the mean of the participant group to which the value belonged. No outliers were identified on any of the dependent variables.

For each of the continuous outcome variables (problem recognition, perceived need for treatment, perceived level of distress, perceived acceptability, and perceived prevalence) separate 3 [Gender condition (between-subjects variable): non-binary vs. male vs. female] × 3 [ED type (repeated measures variable): BN vs. AN vs. BED] mixed ANOVAs were computed. Significant main effects were followed by Tukey-corrected pairwise comparisons. For the categorical outcome variables (i.e., accurately identify the problem described in the vignette as disordered eating), separate Chi-square analyses were conducted for each ED type.

## Results

### Sample characteristics

Demographic characteristics across the non-binary, male, and female gender conditions are presented in Table 1. Chi-square tests and one-way ANOVAs indicated that the participants in the three conditions did not differ significantly on any demographic variable.

**Table 1:** Demographic characteristics across participant groups

	Non-binary (n = 75)	Male (n = 75)	Female (n = 75)	*F* /${\chi }^{2}$	*p*
Age, M (SD)	29.1 (4.95)	30.2 (8.55)	29.3 (8.64)	0.453	0.636
Gender, *n* (%)					
Female	50 (66.7)	54 (72.0)	54 (72.0)	0.680	0.712
Male	25 (33.3)	21 (28.0)	21 (28.0)		
Marital status, *n* (%)					
Married	39 (52.0)	40 (53.3)	35 (46.7)	11.54	0.317
Widowed	0 (0.0)	1 (1.3)	0 (0.0)		
Divorced	5 (6.7)	1 (1.3)	4 (5.3)		
Never married	24 (32.0)	25 (33.3)	28 (37.3)		
Unmarried couple	3 (4.0)	8 (10.7)	6 (8.0)		
Separated	4 (5.3)	0 (0.0)	2 (2.7)		
Race, *n* (%)					
White	64 (85.3)	70 (93.3)	71 (97.5)	7.74	0.459
Black	2 (2.7)	1 (1.3)	1 (1.3)		
Indigenous	5 (6.7)	1 (1.3)	1 (1.3)		
Other	4 (5.3)	3 (4.0)	2 (2.7)		
Ethnicity, *n* (%)					
Hispanic/Latino	37 (49.3)	34 (45.3)	31 (41.3)	0.968	0.616
Non- Hispanic/Latino	38 (50.7)	41 (54.7)	44 (58.7)		
Education, *n* (%)					
Grade school	1 (1.3)	0 (0.0)	0 (0.0)	6.30	0.390
High school	0 (0.0)	3 (4.0)	1 (1.3)		
College	57 (76.0)	53 (70.7)	59 (78.7)		
Graduate school	17 (22.7)	19 (25.3)	15 (20.0)		
Sexual orientation, *n* (%)					
Heterosexual	64 (85.3)	68 (90.7)	69 (92.0)	11.31	0.334
Bisexual	5 (6.7)	7 (9.3)	3 (4.0)		
Lesbian	1 (3.1)	0 (0.0)	2 (2.7)		
Gay	3 (4.0)	0 (0.0)	1 (1.3)		
Questioning	2 (2.7)	0 (0.0)	0 (0.0)		
SES, *n* (%)					
Lower	1 (1.3)	0 (0.0)	1 (1.3)	3.96	0.861
Lower-Middle	6 (8.0)	3 (4.0)	4 (5.3)		
Middle	34 (45.3)	37 (49.3)	33 (44.0)		
Upper-Middle	30 (40.0)	27 (36.0)	31 (41.3)		
Upper	4 (5.3)	8 (10.7)	6 (8.0)		

### Problem recognition

Contrary to the study hypothesis, there was no main effect of gender condition on problem recognition, *F* = 1.72, *p* = 0.18. The gender condition by ED type interaction was also non-significant, *p* > 0.05. There was a main effect of ED type,* F* = 18.74, *p* < 0.001. Participants, regardless of gender condition, rated the issue described in the BN vignette as more likely to be a problem than the issue described in the BED vignette. Participants also rated the issue described in the AN vignette as more likely to be a problem than the issue described in the BED vignette. Problem recognition did not differ significantly between the AN and BN vignettes.

### ED recognition

For BN, the proportion of participants who classified the issue described in the vignette as some form of eating pathology (versus other) did not differ significantly across gender conditions, χ^2^ (2) = 2.98, *p* = 0.23. Similarly, for AN, the proportion of participants who classified the issue described in the vignette as some form of eating pathology (versus other) did not differ significantly across gender conditions, χ^2^ (2) = 1.97, *p* = 0.37. For BED, the proportion of participants who classified the issue described in the vignette as some form of eating pathology (versus other) did not differ significantly across gender conditions, χ^2^ (2) = 1.63, *p* = 0.44.

### Perceived need for treatment

Contrary to the study hypothesis, there was no main effect of gender condition on perceived need for treatment, *F* = 0.82, *p* = 0.44. The gender condition by ED type interaction was also non-significant, *p* > 0.05. There was a main effect of ED type, *F* = 16.64, *p* < 0.001. Participants, regardless of the gender condition they were assigned to, were more likely to refer the individual described for treatment in the AN vignette than in the BN and BED vignettes. Perceived need for treatment did not differ significantly between the BN and BED vignettes.

### Perceived level of distress

Contrary to the study hypothesis, there was no main effect of gender condition on perceived level of distress, *F* = 0.59, *p* = 0.56. The gender condition by ED type interaction was also non-significant, *p* > 0.05. There was a main effect of ED type,* F* = 10.29, *p* < 0.001. Participants, regardless of the gender condition they were assigned to, rated a higher perceived level of distress for the individual described for the AN vignette than for the BN and BED vignettes. Perceived level of distress did not differ significantly between the BN and BED vignettes.

### Acceptability

Contrary to the study hypothesis, there was no main effect of gender condition on acceptability, *F* = 0.03, *p* = 0.98. The gender condition by ED type interaction was also non-significant, *p* > 0.05. There was also no main effect of ED type, *F* = 1.14, *p* = 0.32.

### Perceived prevalence

Contrary to the study hypothesis, there was no main effect of gender condition on perceived prevalence, *F* = 1.04, *p* = 0.36. The gender condition by ED type interaction was significant, *F* = 2.34, *p* = 0.05. For the BN vignettes, perceived prevalence was significantly higher for the female condition than for the male and non-binary conditions. For the AN vignettes, perceived prevalence was significantly higher for the female and non-binary conditions than for the male condition. There were no significant differences between gender conditions for the BED vignettes. There was also a main effect of ED type, *F* = 7.72, *p* < 0.001*.*

Participants, regardless of gender condition, rated a higher perceived prevalence for the BN vignette than for the AN vignette. Perceived prevalence did not differ significantly between the AN and BED vignettes or the BN and BED vignettes.

## Discussion

The purpose of this study was to examine whether ED recognition, perceived need for treatment, perceived distress, perceived acceptability, and perceived prevalence differed depending on the gender of the individual with the ED. We found that participants rated a higher prevalence of AN in women and non-binary individuals than men as well as a higher prevalence of BN in women than men and non-binary individuals. However, varying the gender of the individual described in the vignettes did not influence ED recognition, perceived need for treatment, perceived distress, perceived acceptability, or perceived prevalence. In addition, we found significant differences in the rates of ED recognition, perceived need for treatment, perceived distress, and perceive prevalence across the three different EDs.

Participants rated a higher prevalence of AN in women and non-binary individuals than men, as well as a higher prevalence of BN in women than men and non-binary individuals. This finding may in part be because of differences in the presentation of EDs in men vs. women [23]. The ED presentation in men is usually muscularity-oriented [24], with men focusing on attaining a more muscular and lean body type and experiencing a low drive for thinness [23]. Men are also more likely to use exercise as a compensatory behavior than women [23]. However, DSM descriptions of EDs are biased towards a female presentation (i.e., focusing on drive for thinness and body dissatisfaction instead of a desire to be more muscular). Until the most recent edition, the DSM even listed amenorrhea as a required criterion for AN, implying that only people who menstruate could have an ED. Furthermore, many current ED assessment tools are over-reliant on items that evaluate stereotypically feminine indicators of ED pathology [25] and EDs are depicted in the media as affecting mainly women [5, 18]. All of these factors may contribute to the perception of an AN and BN as female disorders, reinforcing the idea that it is not likely for men and/or non-binary individuals to have an ED. In non-binary individuals, disordered eating behaviors may be used to attain a body type that is in line with their identified gender [15]. The lower perceived prevalence of AN and BN in men and/or non-binary individuals may also relate to the higher rates of social stigma of EDs seen in non-female populations [17, 19]. The higher perceived prevalence of AN in females and non-binary individuals and the higher prevalence of BN in females is problematic because EDs also affect males and non-binary individuals. As of 2008, the NIMH estimated that roughly one million men struggle with EDs [23] and male EDs make up at least 25% of all AN and BN cases [12]. Furthermore, more and more men are seeking help or being identified in treatment [23]. It is thought that these estimated ED prevalence rates for men are underestimated due to the stigma associated with ED pathology in men and the potential for minimization of symptoms [28]. In contrast, we found that gender did not influence the perceived prevalence of BED. This may be in part be because the gender ratio for BED prevalence is less skewed than that of AN and BN [11].

Results suggest that the gender of the individual portrayed in the vignette did not influence problem recognition, ED recognition, perceived need for treatment, perceived level of distress, or acceptability. Our finding that problem and ED recognition did not differ among gender conditions stands in contrast to other research where participants were more able to identify AN and BN if the vignette target was a woman than a man [7]. This may be because of the difference in demographics of the participant samples; Blackstone and colleagues surveyed only college students. In contrast, our sample consisted of participants with a wider age range, a majority of who had already graduated college and were in the workforce. EDs are more common on college campuses than the larger community [29], which may impact the extent to which college students are able to identify EDs in different populations.

We found differences in the rates of ED recognition, perceived need for treatment, perceived distress, and perceived prevalence across the three different EDs. Participants were more likely to recognize BN and AN as a problem than BED. This finding may be related to low BED awareness, since it is a newer formal diagnosis in the latest version of the DSM [11]. Furthermore, AN and BN symptoms most closely fit the stereotypical image of a thin or underweight individual. In contrast, BED does not fit the perception of a stereotypic ED presentation (e.g., being underweight). Our findings are partially in line with past research where AN was more recognizable than BN and BED [1] as well as another study where BN was more recognizable than AN and BED [7]. We found higher perceived need for treatment and perceived level of distress for AN than BED and BN. This is in line with past research that found higher perceived need for treatment for AN than BED and BN [1]. Participants may have rated AN to be more distressing than BN and BED and were more likely to refer AN to treatment than BN and BED due to the severity of AN. For example, the mortality rate in AN is higher than in BED and one of the highest across all psychiatric illnesses [30]. Participants also rated a higher perceived prevalence for BN than for AN. This may be because population lifetime prevalence of BN (1.9% for women and 0.6% for men) is in actuality higher than that of AN (1.4% for women and 0.2% for men) [31]. In contrast, perceived acceptability did not differ across the three EDs, with participants describing acceptability as, on average, being “unsure if it would be too bad to have these problems” for all three EDs.

### Strengths and limitations

One potential limitation is the homogeneity of the sample. Most participants were young White cisgender heterosexual females who were highly educated (e.g., completed at least college). Future studies might expand on these findings or arrive at different results if the study is distributed to a larger and less homogenous group. In addition, the vignettes did not portray any internal dialogue surrounding the disordered eating behaviors such as weight or body image. Although this was intentional as the focus of the study was on recognition of observable behaviors, thoughts related to food and body image are integral aspect of EDs. However, ED cognitions are rarely noticeable to others. Recognition and perceived need for treatment may have improved if the vignettes included the internal experiences consistent with AN, BN, and BED. Relatedly, the vignettes did not greatly vary between conditions to remain consistent. However, not varying the vignettes between conditions may pose as a limitation because of the differences in presentation of EDs in different genders [15, 16]. Another important limitation to consider is that there was no validation conducted for the measures used to assess problem/ED recognition, perceived need for treatment, perceived level of distress, acceptability, and perceived prevalence for these vignettes. However, the measures we used have been used in other studies [1, 5, 6]. Nevertheless, the wording of the questions may have been somewhat vague and may have been misinterpreted by participants. Finally, in describing the non-binary condition, we only stated that the individual identified as non-binary and used they/them/their pronouns, but did not have a definition provided to participants as to what nonbinary means. Some participants may not have known what a nonbinary identity is, particularly because most of the participants in our sample were cisgender.

### What is already known on this subject?

The general population has several stereotypes about who may physically look like they have an eating disorder. Previous studies have identified various stereotypes, such as race/ethnicity and weight stereotypes, regarding eating disorder recognition, perceived need for treatment, perceived distress, perceived acceptability, and/or perceived prevalence. There is limited evidence regarding gender stereotypes, with findings from one study suggesting that college students are more able to identify anorexia nervosa and bulimia in women than men.

### What this study adds?

Findings suggest that individuals believe that anorexia nervosa is more prevalent in women and non-binary individuals than men, and that bulimia nervosa is more prevalent in women than non-binary individuals and men.

## Data Availability

Data are available upon request.

## Appendix

This appendix consists of the vignettes that participants read.

## Male

### BN

Ben is 20 years old and identifies as male

**Monday****: **Ben woke up, showered, and got dressed. For breakfast, he had a banana and cereal. After breakfast, he went to his classes at his college. During lunch, he ate a slice of pizza and a salad. After school, Ben spent time with his friends and then went home. When he got home, he ate dinner with his roommates, which included roasted chicken and roasted potatoes with broccoli: his favorite. He had seconds and thirds. His roommate bought cookies. He had one and they tasted so good. Before he knew it, he had eaten 4.

**Tuesday: **He woke up early and went for a long 7-mile run. He had coffee for breakfast. Ben went to school. During lunch he ate a big salad, soda, and an apple. After classes, Ben had track practice. When he got home, he was tired, so he took a nap. His roommate woke his up for dinner, and he had pasta. He later did his homework and went to sleep.

**Wednesday:** He woke up and got dressed. He had a bagel and fruit for breakfast. He had a sandwich he brought from home for classes. When he got home, he found nobody else was home, so he watched a movie and had two big bags of chips. He made a pizza for dinner. He said he was just going to have two pieces, and that he would leave the rest for his roommates, but he just couldn’t stop. He ate half of the pizza.

**Thursday:** He woke up early and went to the gym for two hours. He didn’t have time to shower or eat breakfast afterwards, and he was almost late for school. For lunch, he had a granola bar and a nectarine. After classes, he had track club. Then he went shopping with his roommate. His roommate made pasta for dinner, and he had two big plates of pasta. His friend brought over brownies for dessert, which he and his roommates had with vanilla ice cream. He had seconds and thirds. He then watched TV and did his homework. While doing his homework, he felt a bit too full of dinner, so he drank a laxative tea.

**Friday: **He woke up and went for a 6-mile run. He then got dressed and had cereal for breakfast. He went to school and was excited that it was Friday. During lunch he had tuna salad with fruit and crackers. After classes, he went to get pizza with his friends. When he came home, his roommate had made his favorite casserole, so he had a little of that too. He went for a long walk with his roommate after dinner.

### AN

Mark is 20 years old and identifies as male

**Monday: **Mark woke up and took a shower. Mark tried on three different outfits before choosing what he was going to wear. He fixed his hair twice before leaving for classes at his college. For breakfast, he had a banana. Mark went to school. During lunch he ate three rice cakes and drank an apple juice. After school, Mark had soccer practice for two hours and then went home. When he got home, he took a shower and then did his homework. For dinner, Mark ate salad and a plain baked potato. He watched TV for two hours and then went to bed.

**Tuesday:** He woke up and took a shower. Mark tried on several different shirts before choosing which one he was going to wear. He spent half an hour gelling his hair. He didn’t have time for breakfast, so he drank some orange juice. Mark went to school. During lunch he ate some pretzels, a diet soda, and a pear. After classes, Mark had soccer practice for two hours and a one-hour meeting for his fraternity. When he got home, he drank some water and took a shower. Then he did his homework. For dinner, Mark ate a small cup of vegetable soup with crackers and drank a diet soda. He studied for a test for two hours, picked out his clothes for the next day for half an hour and then went to bed.

**Wednesday:** He woke up and took a shower and got dressed. He fixed his hair for twenty minutes. He had a piece of toast and some apple juice for breakfast. Mark had a test in the morning on which he felt he did poorly on and was upset. Instead of eating lunch, he did his homework. After classes, Mark had soccer practice for two hours and then went home. When he got home, he drank some diet soda. He then took a shower and watched TV. For dinner, Mark ate some crackers, a salad, and drank some water. Mark watched TV, played video games with his friend, and then went to bed.

**Thursday:** He woke up and took a shower. He got dressed and ate an apple for breakfast. He went to school. For lunch, he had a granola bar, an orange, and some diet soda. After classes, he had soccer practice and then went home. When he got home, he didn’t eat anything and just took a shower. He then watched some TV. For dinner, Mark drank some water and had a bag of chips. He then finished his homework and had some raisins before going to bed.

**Friday: **He woke up and took a shower. He got dressed and gelled his hair. For breakfast he had a grapefruit. He went to school and found out he did poorly on the test he took on Wednesday and was upset. During lunch he ate an egg salad and some grape juice. After classes, Mark had soccer club for two hours and then went home. He went home and took a shower. For dinner he ate some black beans and rice. He then went to the movies with his friends.

### BED

Ethan is 20 years old and identifies as male

**Monday:** Ethan woke up late and had to rush to class. He didn’t have time to eat breakfast. Because he was planning on doing homework for lunch, he had to eat a few granola bars for lunch. For dinner, his roommate made lasagna, which he had with a salad. He had seconds and thirds. After he finished eating dinner, he had a bowl of ice cream. After dinner, he tried to do his homework but couldn’t concentrate, so he went to sleep early instead.

**Tuesday:** Ethan woke up early and made pancakes for himself and his roommates for breakfast. He ate breakfast with his roommates, and then went to school. During lunch, he bought a salad from the dining hall. After classes, he spent a few hours in the library studying. He finished all that was left of yesterday’s lasagna and salad, as well as the leftover ice cream. After dinner, Ethan watched a movie. He had some fruit and went to bed.

**Wednesday:** He woke up and took a shower and got dressed. He still felt full, so he skipped breakfast. For lunch, he was famished so Ethan bought a hamburger and fries from the cafeteria. His girlfriend broke up with his at school today, and Ethan was upset all day. He stopped at the drug store on his way back home and got a four chocolate bars, a pint of Ben & Jerry’s ice cream, and some bags of chips. He spent the evening watching movies and eating all the chocolate, ice cream, and chips.

**Thursday:** Ethan woke up feeling sorry for himself after the breakup, but also telling himself he should move on. He had a coffee for breakfast. He had a test in one of his classes, which he was happy to get over with. For lunch, he got a big salad with a soda and crackers. He had a club meeting after classes. He had chicken, potatoes, and broccoli for dinner. He then tried to go to sleep but couldn’t fall asleep. He got up and had milk, a banana with peanut butter, and a bowl of ice cream to help his fall asleep. He couldn’t stop at a bowl, so he finished the whole carton. Then he fell asleep.

**Friday:** He woke up and took a shower and got dressed. He skipped breakfast because he still felt too full from the night before. He went to school and found out he did well on yesterday’s test. His girlfriend also told him she wanted to get back together. He was very happy for the rest of the day. He had pizza for lunch. After classes, he and his girlfriend went to the movies and a nice Italian restaurant for dinner.

## Cameron is 20 years old and identifies as non-binary

**Monday:** Cameron woke up, showered, and got dressed. For breakfast, they had a banana and cereal. After breakfast, they went to their classes at her college. During lunch, they ate a slice of pizza and a salad. After school, Cameron spent time with their friends and then went home. When they got home, they ate dinner with their roommates, which included roasted chicken and roasted potatoes with broccoli: their favorite. They had seconds and thirds. Their roommate baked cookies. They had one and they tasted so good. Before they knew it, they had eaten 4.

**Tuesday:** They woke up early and went for a long 7-mile run. They had coffee for breakfast. Cameron went to school. During lunch they ate a big salad, soda, and an apple. After classes, Cameron had track practice. When they got home, they were tired, so they took a nap. Their roommate woke them up for dinner, and they had pasta. They later did their homework and went to sleep.

**Wednesday: **They woke up and got dressed. They had a bagel and fruit for breakfast. They had a sandwich they brought from home for classes. When they got home, they found nobody else was home, so they watched a movie and had two big bags of chips. They made a pizza for dinner. They said they were just going to have two pieces, and that they would leave the rest for their roommates, but they just couldn’t stop. They ate half of the pizza.

**Thursday: **They woke up early and went to the gym for two hours. They didn’t have time to shower or eat breakfast afterwards, and they were almost late for school. For lunch, they had a granola bar and a nectarine. After classes, they had track club. Then they went shopping with their roommate. Their roommate made pasta for dinner, and they had two big plates of pasta. Their friend baked brownies for dessert, which they and their roommates had with vanilla ice cream. They had seconds and thirds. They then watched TV and did their homework. While doing their homework, they felt a bit too full of dinner, so they drank a laxative tea.

**Friday:** They woke up and went for a 6-mile run. They then got dressed and had cereal for breakfast. They went to school and were excited that it was Friday. During lunch they had tuna salad with fruit and crackers. After classes, they went to get pizza with their friends. When they came home, their roommate had made their favorite casserole, so they had a little of that too. They went for a long walk with their roommate after dinner.

### AN

Jordan is 20 years old and identifies as non-binary

**Monday: **Jordan woke up and took a shower. Jordan tried on three different outfits before choosing what they were going to wear. They fixed their hair twice before leaving for classes at her college. For breakfast, they had a banana. Jordan went to school. During lunch they ate three rice cakes and drank an apple juice. After school, Jordan had soccer practice for two hours and then went home. When they got home, they took a shower and then did their homework. For dinner, Jordan ate salad and a plain baked potato. They watched TV for two hours and then went to bed.

**Tuesday:** They woke up and took a shower. Jordan tried on several different shirts before choosing which one they were going to wear. They spent half an hour fixing their hair. They didn’t have time for breakfast, so they drank some orange juice. Jordan went to school. During lunch they ate some pretzels, a diet soda, and a pear. After classes, Jordan had soccer practice for two hours and a one-hour meeting for a club. When they got home, they drank some water and took a shower. Then they did their homework. For dinner, Jordan ate a small cup of vegetable soup with crackers and drank a diet soda. They studied for a test for two hours, picked out their clothes for the next day for half an hour and then went to bed.

**Wednesday:** They woke up and took a shower and got dressed. They did their hair for twenty minutes. They had a piece of toast and some apple juice for breakfast. Jordan had a test in the morning which they felt they did poorly on and were upset. Instead of eating lunch, they did their homework. After classes, Jordan had soccer practice for two hours and then went home. When they got home, they drank some diet soda. They then took a shower and watched TV. For dinner, Jordan ate some crackers, a salad, and drank some water. Jordan watched TV, talked on the phone with their friend, and then went to bed.

**Thursday: **They woke up and took a shower. They got dressed and ate an apple for breakfast. They went to school. For lunch, they had a granola bar, an orange, and some diet soda. After classes, they had soccer practice and then went home. When they got home, they didn’t eat anything and just took a shower. They then watched some TV. For dinner, Jordan drank some water and had a bag of chips. They then finished their homework and had some raisins before going to bed.

**Friday: **They woke up and took a shower. They got dressed and fixed their hair. For breakfast they had a grapefruit. They went to school and found out they did poorly on the test they took on Wednesday and was upset. During lunch they ate an egg salad and some grape juice. After classes, Jordan had soccer club for two hours and then went home. They went home and took a shower. For dinner they ate some black beans and rice. They then went to the movies with their friends.

### BN

Sam is 20 years old and identifies as non-binary

**Monday: **Sam woke up late and had to rush to class. They didn’t have time to eat breakfast. Because they were planning on doing homework during lunch, they had to eat a few granola bars for lunch. For dinner, their roommate made lasagna, which they had with a salad. They had seconds and thirds. After they finished eating dinner, they had a bowl of ice cream. After dinner, they tried to do their homework but couldn’t concentrate, so they went to sleep early instead.

**Tuesday:** Sam woke up early and made pancakes for themself and their roommates for breakfast. Sam ate breakfast with their roommates, and then went to school. During lunch, they bought a salad from the dining hall. After classes, they spent a few hours in the library studying. They finished all that was left of yesterday’s lasagna and salad, as well as the leftover ice cream. After dinner, Sam watched a movie. They had some fruit and went to bed.

**Wednesday:** They woke up and took a shower and got dressed. They still felt full, so they skipped breakfast. For lunch, they were famished so Sam bought a hamburger and fries from the cafeteria. Their partner broke up with them at school today, and Sam was upset all day. They stopped at the drug store on their way back home and got a four chocolate bars, a pint of Ben & Jerry’s ice cream, and some bags of chips. They spent the evening watching movies and eating all the chocolate, ice cream, and chips.

**Thursday:** Sam woke up feeling sorry for themself after the breakup, but also telling themself they should move on. They had a latte for breakfast. They had a test in one of their classes, which they were happy to get over with. For lunch, they got a big salad with a soda and crackers. They had a club meeting after classes. They had chicken, potatoes, and broccoli for dinner. They then tried to go to sleep but couldn’t fall asleep. They got up and had milk, a banana with peanut butter, and a bowl of ice cream to help them fall asleep. They couldn’t stop at a bowl, so they finished the whole carton. Then they fell asleep.

**Friday: **They woke up and took a shower and got dressed. They skipped breakfast because they still felt too full from the night before. They went to school and found out they did well on yesterday’s test. Their partner also told them they wanted to get back together. They were very happy for the rest of the day. They had pizza for lunch. After classes, they and their partner went to the movies and a nice Italian restaurant for dinner.

## Female

### BED

Ellen is 20 years old and identifies as female

**Monday:** Ellen woke up late and had to rush to class. She didn’t have time to eat breakfast. Because she was planning on doing homework for lunch, she had to eat a few granola bars for lunch. For dinner, her roommate made lasagna, which she had with a salad. She had seconds and thirds. After she finished eating dinner, she had a bowl of ice cream. After dinner, she tried to do her homework but couldn’t concentrate, so she went to sleep early instead.

**Tuesday:** Ellen woke up early and made pancakes for herself and her roommates for breakfast. She ate breakfast with her roommates, and then went to school. During lunch, she bought a salad from the dining hall. After classes, she spent a few hours in the library studying. She finished all that was left of yesterday’s lasagna and salad, as well as the leftover ice cream. After dinner, Ellen watched a movie. She had some fruit and went to bed.

**Wednesday:** She woke up and took a shower and got dressed. She still felt full, so she skipped breakfast. For lunch, she was famished so Ellen bought a hamburger and fries from the cafeteria. Her boyfriend broke up with her at school today, and Ellen was upset all day. She stopped at the drug store on her way back home and got a four chocolate bars, a pint of Ben & Jerry’s ice cream, and some bags of chips. She spent the evening watching movies and eating all the chocolate, ice cream, and chips.

**Thursday:** Ellen woke up feeling sorry for herself after the breakup, but also telling herself she should move on. She had a latte for breakfast. She had a test in one of her classes, which she was happy to get over with. For lunch, she got a big salad with a soda and crackers. She had a club meeting after classes. She had chicken, potatoes, and broccoli for dinner. She then tried to go to sleep but couldn’t fall asleep. She got up and had milk, a banana with peanut butter, and a bowl of ice cream to help her fall asleep. She couldn’t stop at a bowl, so she finished the whole carton. Then she fell asleep.

**Friday:** She woke up and took a shower and got dressed. She skipped breakfast because she still felt too full from the night before. She went to school and found out she did well on yesterday’s test. Her boyfriend also told her he wanted to get back together. She was very happy for the rest of the day. She had pizza for lunch. After classes, she and her boyfriend went to the movies and a nice Italian restaurant for dinner.

### AN

Meredith is 20 years old and identifies as female

**Monday: **Meredith woke up and took a shower. Meredith tried on three different outfits before choosing what she was going to wear. She fixed her hair twice before leaving for classes at her college. For breakfast, she had a banana. Meredith went to school. During lunch she ate three rice cakes and drank an apple juice. After school, Meredith had soccer practice for two hours and then went home. When she got home, she took a shower and then did her homework. For dinner, Meredith ate salad and a plain baked potato. She watched TV for two hours and then went to bed.

**Tuesday:** She woke up and took a shower. Meredith tried on several different shirts before choosing which one she was going to wear. She spent half an hour curling her hair. She didn’t have time for breakfast, so she drank some orange juice. Meredith went to school. During lunch she ate some pretzels, a diet soda, and a pear. After classes, Meredith had soccer practice for two hours and a one-hour meeting for her sorority. When she got home, she drank some water and took a shower. Then she did her homework. For dinner, Meredith ate a small cup of vegetable soup with crackers and drank a diet soda. She studied for a test for two hours, picked out her clothes for the next day for half an hour and then went to bed.

**Wednesday:** She woke up and took a shower and got dressed. She did her hair for twenty minutes. She had a piece of toast and some apple juice for breakfast. Meredith had a test in the morning on which she felt she did poorly on and was upset. Instead of eating lunch, she did her homework. After classes, Meredith had soccer practice for two hours and then went home. When she got home, she drank some diet soda. She then took a shower and watched TV. For dinner, Meredith ate some crackers, a salad, and drank some water. Meredith watched TV, talked on the phone with her friend, and then went to bed.

**Thursday:** She woke up and took a shower. She got dressed and ate an apple for breakfast. She went to school. For lunch, she had a granola bar, an orange, and some diet soda. After classes, she had soccer practice and then went home. When she got home, she didn’t eat anything and just took a shower. She then watched some TV. For dinner, Meredith drank some water and had a bag of chips. She then finished her homework and had some raisins before going to bed.

**Friday: **She woke up and took a shower. She got dressed and curled her hair. For breakfast she had a grapefruit. She went to school and found out she did poorly on the test she took on Wednesday and was upset. During lunch she ate an egg salad and some grape juice. After classes, Meredith had soccer club for two hours and then went home. She went home and took a shower. For dinner she ate some black beans and rice. She then went to the movies with her friends.

### BN

Olivia is 20 years old and identifies as female

**Monday****: **Olivia woke up, showered, and got dressed. For breakfast, she had a banana and cereal. After breakfast, she went to her classes at her college. During lunch, she ate a slice of pizza and a salad. After school, Olivia spent time with her friends and then went home. When she got home, she ate dinner with her roommates, which included roasted chicken and roasted potatoes with broccoli: her favorite. She had seconds and thirds. Her roommate baked cookies. She had one and they tasted so good. Before she knew it, she had eaten 4.

**Tuesday****: **She woke up early and went for a long 7-mile run. She had coffee for breakfast. Olivia went to school. During lunch she ate a big salad, soda, and an apple. After classes, Olivia had track practice. When she got home, she was tired, so she took a nap. Her roommate woke her up for dinner, and she had pasta. She later did her homework and went to sleep.

**Wednesday****: **She woke up and got dressed. She had a bagel and fruit for breakfast. She had a sandwich she brought from home for classes. When she got home, she found nobody else was home, so she watched a movie and had two big bags of chips. She made a pizza for dinner. She said she was just going to have two pieces, and that she would leave the rest for her roommates, but she just couldn’t stop. She ate half of the pizza.

**Thursday****: **She woke up early and went to the gym for two hours. She didn’t have time to shower or eat breakfast afterwards, and she was almost late for school. For lunch, she had a granola bar and a nectarine. After classes, she had track club. Then she went shopping with her roommate. Her roommate made pasta for dinner, and she had two big plates of pasta. Her friend baked brownies for dessert, which she and her roommates had with vanilla ice cream. She had seconds and thirds. She then watched TV and did her homework. While doing her homework, she felt a bit too full of dinner, so she drank a laxative tea.

**Friday****: **She woke up and went for a 6-mile run. She then got dressed and had cereal for breakfast. She went to school and was excited that it was Friday. During lunch she had tuna salad with fruit and crackers. After classes, she went to get pizza with her friends. When she came home, her roommate had made her favorite casserole, so she had a little of that too. She went for a long walk with her roommate after dinner.
